# APEX - the Hyperspectral ESA Airborne Prism Experiment

**DOI:** 10.3390/s8106235

**Published:** 2008-10-01

**Authors:** Klaus I. Itten, Francesco Dell'Endice, Andreas Hueni, Mathias Kneubühler, Daniel Schläpfer, Daniel Odermatt, Felix Seidel, Silvia Huber, Jürg Schopfer, Tobias Kellenberger, Yves Bühler, Petra D'Odorico, Jens Nieke, Edoardo Alberti, Koen Meuleman

**Affiliations:** 1 Remote Sensing Laboratories, University of Zurich – Irchel, Winterhurerstrasse 190, CH-8057 Zurich, Switzerland; E-mails: klaus.itten@geo.uzh.ch; andreas.hueni@geo.uzh.ch; mathias.kneubuehler@geo.uzh.ch; daniel.schlaepfer@geo.uzh.ch; dani.odermatt@geo.uzh.ch; felix.seidel@geo.uzh.ch; silvia.huber@geo.uzh.ch; juerg.schopfer@geo.uzh.ch; tobias.kellenberger@geo.uzh.ch; yves.buehler@geo.uzh.ch; petra.dodorico@geo.uzh.ch; jens.nieke@geo.uzh.ch; edoardo.alberti@geo.uzh.ch; 2 VITO – Flemish Institute for Technological Research, Boeretang, 200 B-2400 Mol, Belgium; E-mail: koen.meuleman@vito.be

**Keywords:** Hyperspectral, pushbroom, imaging spectrometer

## Abstract

The airborne ESA-APEX (Airborne Prism Experiment) hyperspectral mission simulator is described with its distinct specifications to provide high quality remote sensing data. The concept of an automatic calibration, performed in the Calibration Home Base (CHB) by using the Control Test Master (CTM), the In-Flight Calibration facility (IFC), quality flagging (QF) and specific processing in a dedicated Processing and Archiving Facility (PAF), and vicarious calibration experiments are presented. A preview on major applications and the corresponding development efforts to provide scientific data products up to level 2/3 to the user is presented for limnology, vegetation, aerosols, general classification routines and rapid mapping tasks. BRDF (Bidirectional Reflectance Distribution Function) issues are discussed and the spectral database SPECCHIO (Spectral Input/Output) introduced. The optical performance as well as the dedicated software utilities make APEX a state-of-the-art hyperspectral sensor, capable of (a) satisfying the needs of several research communities and (b) helping the understanding of the Earth's complex mechanisms.

## 1. Introduction

Early hyperspectral airborne experiments in Europe in the late 80s, EISAC (European Imaging Spectrometry Airborne Campaign), and especially the deployment of AVIRIS (Airborne Visible/Infrared Imaging Spectrometer) [1] in the MAC-Europe campaign (Multi Aircraft Campaign) in 1991, which consolidated a sound research community, showed that a European instrument and an according industrial development was required for securing continuation in hyperspectral research.

Hyperspectral imaging spectrometers integrate imaging and spectroscopy in a single system, providing a series of contiguous and narrow spectral channels for the study of Earth surface materials in the solar-reflected region of the electromagnetic spectrum, i.e. between 380 nm and 2500 nm.

Even though a few systems were acquired from overseas, namely CASI (Compact Airborne Spectrographic Imager) [2], GERIS (Geophysical Environment Research Imaging Spectrometer) [3] and DAIS (Digital Airborne Imaging Spectrometer) [4], which provided state of the art data, it became obvious that ESA (European Space Agency) was in need of a flexible hyperspectral space mission simulator and applications demonstrator covering the full VIS-NIR-SWIR (Visible-Near-Infrared-Shortwave Infrared) wavelength range. The national development of ROSIS (Reflective Optics System Imaging Spectrometer) in Germany was meant to partially serve this purpose. Spectra Vista's Hymap (Hyperspectral Scanner) [5] instrument was leased in the late 90s and early 2000, and AHS (Airborne Hyperspectral System) [6] was used to cover the basic experimental needs of the hyperspectral research community.

The planning for APEX (Airborne Prism Experiment) started in 1993, a formal pre-phase A was granted by ESA in 1995. APEX was then designed and developed under ESA-PRODEX (Programme pour le dévelopement des éxperiments) and co-funded by Switzerland and Belgium. An industrial consortium, in phases C and D under the prime contractor RUAG (Rüstungsunternehmungen AG) Aerospace (Emmen, CH), responsible for the total system and the mechanical components, OIP (Oudenaarde, BE) contributing the spectrometer, and Netcetera (Zurich, CH), responsible for the electronics, built APEX. Remote Sensing Laboratories (RSL, University of Zurich, CH) acts as scientific PI together with the Co-PI VITO (Flemish Institute for Technological Research, Mol, BE). The system is currently in the calibration and test phase (phase D), and will deliver first scientific data to users late in 2008. Fully-fledged flight campaigns are foreseen to start in 2009.

APEX is a flexible airborne hyperspectral mission simulator and calibrator for existing and upcoming or future space missions. It is operating between 380 and 2,500 nm in 313 freely configurable bands, up to 534 bands in full spectral mode. Besides general applications development and research, the system is foreseen, to carry out experiments for e.g. ESA Sentinels II and III [7], the evaluated Explorers FLEX (Fluorescent Explorer) [8] and TRAQ (Tropospheric Composition and Air Quality) [9], the German national initiative ENMAP (Advanced Hyperspectral Mission) [10], and the South African MSMI (Multi Sensor Micro satellite Imager) [11] among others.

## 2. Sensor overview

**Figure 1.**
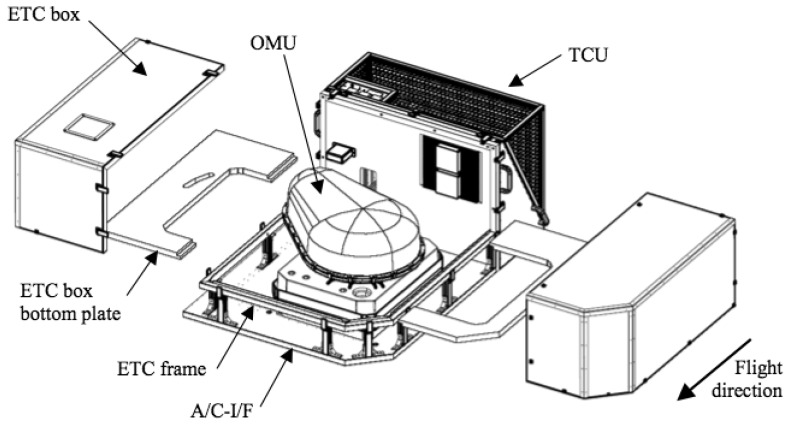
Overview APEX subsystems.

The APEX instrument consists of several sub-units (Figure 1). The optical sub-unit (OSU) is the core element of the instrument including the sensitive optics, properly interfaced with customized front-end electronic (FEE) boards. The OSU is operated on a stabilized platform (STP) in order to dampen all the externally induced vibrations and ensure stable vertical measurements. The platform is controlled by the navigation system, which receives orientation information from an inertial measurement unit (IMU) implemented on the OSU and position signals from a GPS receiver. The orientation and position information are then synchronized with the image data by the control and storage unit (CSU). Each data frame is thus time and day tagged and stored on a hard disk array. This information is finally transferred to the processing and archiving facility (PAF), either over a Gigabit Ethernet or via storage tapes.

The instrument is temperature and pressure stabilized. The opto-mechnical unit (OMU) is enclosed by the environmental thermal control box (ETC). The thermal control unit (TCU) controls the temperature of the OMU cooling circuits and of the ETC box atmosphere. The SWIR (Short Wavelength) detector is directly linked with a dedicated cooling system that keeps its temperature at about -100 °C, thus drastically reducing the thermal noise. The main units are illustrated in Figure 1.

A custom aircraft interface (A/C-I/F) allows the Dornier Do-228 airplane to carry and operate the instrument during the mission phases.

**Figure 2.**
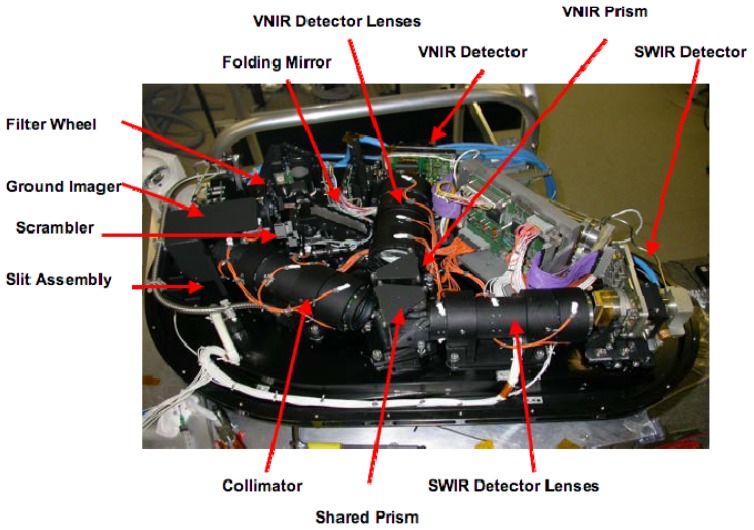
Main elements of the Optical Unit System of the APEX sensor.

A detailed representation of the optical subunit (OSU) is given in Figure 2. This subsystem is composed of the following elements (see Figure 2):

- An entrance window, located underneath the folding mirror.
- One folding mirror, guiding the entering light towards the ground imager.
- A removable polarization scrambler that reduces the polarization sensitivity of the instrument.
- A filter wheel, containing a series of neutral density filters in order to avoid saturation and a series of bandpass filters used in connection with the in-flight calibration facility (IFC).
- A ground imager that images the ground section on the spectrometer rectangular slit, whose dimensions are 0.04 mm × 40 mm.
- A spectrometer section that decomposes the incoming light into its spectral components and re-images the slit image onto two array detectors.

Light enters the spectrometer part through a curved slit and a collimator redirects the light towards a beamsplitter, which separates the VNIR (Visible Near Infrared) wavelengths (380-1000 nm) from the SWIR (Short Wavelength Infrared) wavelengths (950-2,500 nm). The VNIR wavelengths are then dispersed from another face of the beamsplitter/prism and imaged by a CCD (Charged Coupled Device) detector after passing through a customized VNIR optic; the dynamic range of the VNIR is spread over 14 bits. The SWIR wavelengths enter a further prism and are dispersed from a second surface of this prism. A focusing SWIR optic then projects the spectral components onto a CMOS (Complementary Metal Oxide Semiconductor) detector, proving a dynamic range of 12 bits. The pitch size of the CCD detector is 22.5 μm whereas the one of the CMOS is 30 μm. The VNIR array detector can record up to 335 unbinned bands and SWIR 199 unbinned bands. Customized binning patterns can be applied in order to satisfy specific scientific applications.

One of the main features of APEX is providing spatial synchronization of the VNIR and SWIR images, otherwise offered separately from other sensors. This characteristic led to design the instrument with very stringent requirements in order to offer low data uncertainty. Therefore the scanner has been optimized for non-uniformities, mainly caused by the intrinsic nature of the acquisition mechanism and by the non-linear nature of the light. In order to allow users implementing hyperspectral-based applications with a satisfactory radiometric resolution, the APEX bands will provide a high Signal-To-Noise-Ratio (SNR), usually higher than 100. Thanks to its high spectral, spatial and radiometric performances, APEX is a promising instrument that will help researchers in improving significantly the understanding of the Earth.

## 3. Calibration

The APEX calibration concept has been developed in order to offer high quality products in terms of accuracy and tolerance to the user. The calibration strategy is targeted to guarantee an absolute radiometric accuracy of 3%.

The calibration strategy makes use of several utilities:

1. The Calibration Test Master (CTM): a hardware/software utility [12] that automatically performs the on-ground calibration procedures by interfacing APEX with the Calibration Home Base (CHB), a laboratory installation located at DLR (Deutsches Zentrum für Luft-und Raumfahrt) Oberpfaffenhofen (Germany).
2. The In-Flight Calibration facility (IFC): the APEX on-board calibration equipment [13] whose objectives are (a) monitoring the absolute and relative stability of calibration parameters during the operation phases, i.e. the image acquisition, and (b) performing spectral and radiometric in-flight calibration by using a set of customized spectral filters.
3. The Level 0-1 Processor: a software component that includes modules for the transformation of raw image data from digital numbers (DN) to physical units of radiance [14,15], i.e. generating radiometrically, spectrally and geometrically well calibrated, uniform data (Level 1C). The level 0-1 processor has been developed by RSL and is integrated into the APEX Processing and Archiving Facility (PAF).
4. Quality Flags (QF): those are pixel-wise metadata, directly linked to the image data. They provide users with useful information on both sensor performance and product quality. Namely, QF inform users about (a) sensor quality, e.g. bad pixels, bad columns, noise level, saturation, (b) relative and absolute stability of radiometric and spectral calibration parameters and (c) classification information in order to let users employ only the pixels that are consistent with their application (e.g. vegetation, limnology, aerosols, snow, geology, soil).
5. Vicarious Calibration: on-ground campaigns as well as inter-comparison with other sensors data [16] will improve the validation and traceability of the APEX products. RSL owns a number of advanced and state-of-the-art ground equipments, supporting the APEX vicarious calibration approach. The available instrumentation includes the dual-view goniometer system (FIGOS) for bi-directional reflectance distribution function (BRDF) measurements [17], several ASD (Analytical Spectral Devices) spectroradiometers, a certified integrating sphere for absolute radiometric calibration, and Spectralon panels that are tied to a laboratory panel with well known spectral characteristics. Furthermore, the well-established international scientific network gives APEX's science team the chance for sensor data exchanges.
6. Scene-based algorithms: those algorithms are directly applied to the acquired data during post-processing in order to identify smile [18], spatial misregistration [19] and to retrieve spectral response function (SRF) shapes [20] and center wavelengths. In some cases, these procedures can generate absolute coefficients that can eventually be used to improve the respective correction and/or refine the characterization of the detector.

The next sections will give an overview of the core elements of the APEX calibration strategy.

### 3.1. The Calibration Test Master

The APEX calibration strategy focuses on the measurement of several calibration and characterization parameters at selected pixels within the detector area. For this purpose, a calibration test master (CTM) is used [12]. The CTM is a hardware/software facility that optimizes the time needed for the calibration by automatic generation of optical stimuli. Thus no manual action is required, apart from some secondary settings, e.g. switching on/off the light sources. The CTM interfaces APEX with both a laboratory ground facility, i.e. the Calibration Home Base (CHB) in Oberpfaffenhofen (Germany), and an In-Flight Calibration facility (IFC). The instrumentation in both the CHB and the IFC can be controlled remotely via a computer interface, thus enabling automatic measurements.

**Figure 4.**
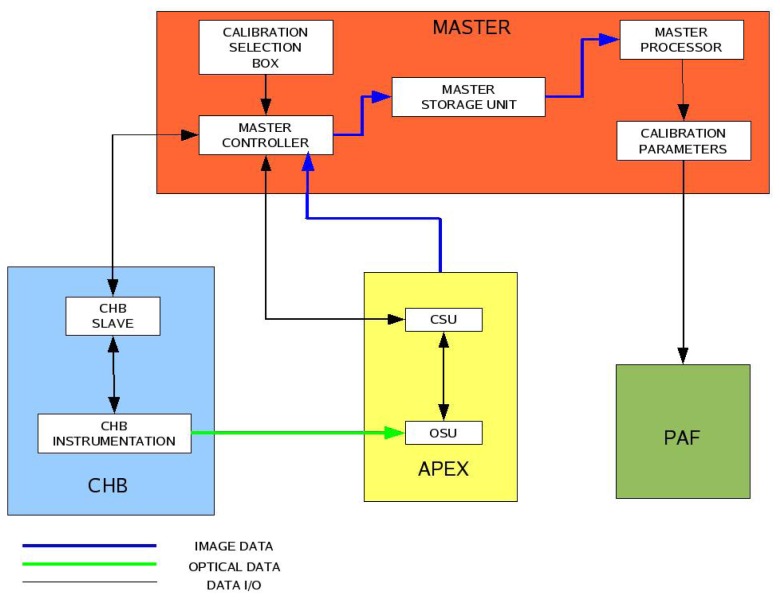
CTM logical working flow. The CTM interfaces APEX, the CHB, and the PAF.

The CTM consists of three main elements (Figure 4):

- *The controller*, which is the core unit of the CTM.
- *The storage unit*, which is partly embedded in APEX and partly located on an external desktop computer.
- *The processor*, whose function is to process all the calibration data.

The CTM controller is embedded in the APEX instrument and sets up all the necessary parameters, i.e. APEX settings (e.g. frame rate, integration time) and calibration facility settings (e.g. monochromator wavelength, integrating sphere lamp intensity) for a particular calibration procedure. Once the setting is completed, the calibration measurements take place and the acquired data are stored in the storage unit. The CTM processor is a complementary software utility, installed on dedicated external hardware, whose goal is to generate the calibration parameters necessary to calibrate the acquired raw data by processing all the data in the CTM storage unit. The Processing and Archiving Facility (PAF) [15] utilizes the calibration parameters provided by the CTM for the level-0 to level-1 processing.

For automated procedures a certain number of sequential sub-requests for both the CHB (e.g. folding mirror height, scan angle, lamp voltage, etc.) and APEX are generated. For each sub-request to be processed by the hardware, the controller generates a well-formatted file, which in turn will be transformed into an electric and/or mechanic signal. The measurements are carried out once the sub-requests have been executed by the relevant hardware. The time needed to process every sub-request has been estimated to be about 5s but this can be reduced if no drastic changes on the setup are necessary. The overall calibration phase therefore requires about one week.

Several units of the laboratory facility can be controlled remotely, e.g. the folding mirror (i.e., linear position, and angular position), the monochromator (e.g., voltage, current, wavelength), the collimator and the integrating sphere (e.g. lamp combination), thus facilitating the automated approach chosen for the CTM.

**Figure 5.**
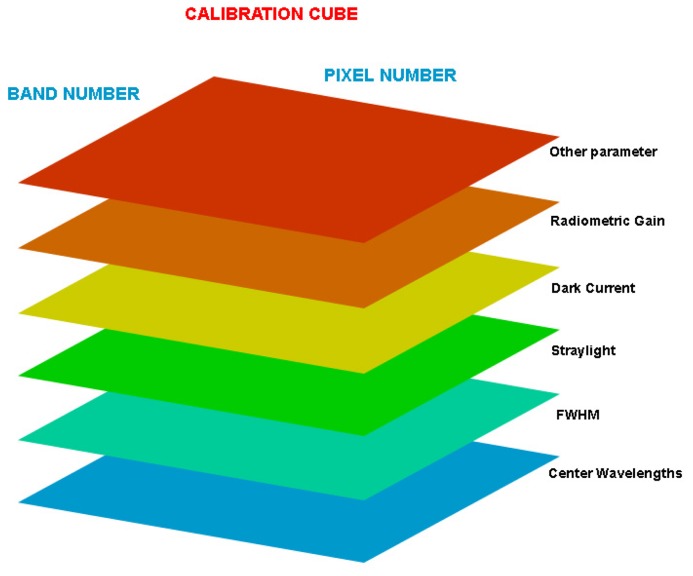
Visualization of a Calibration Cube.

The CTM activities generate a series of information that need to be processed and partly stored. The primary goal is the provision of calibration parameters compiled into the so-called calibration cubes that are used during level0-1 processing. The calibration cube (Figure 5) is a three-dimensional matrix where each of its layers represents a calibration parameter. A layer has the same dimensions as the detector dimensions when it is operated in the un-binned configuration. The third dimension of the cube is formed by the calibration parameters.

In order to distinguish between external calibration sources, e.g. the CHB, and internal calibration sources, e.g. the IFC, another calibration cube is generated containing the IFC measurements. Consequently, four calibration cubes are produced:

- The VNIR calibration cube.
- The SWIR calibration cube.
- The IFC-CHB calibration cubes (VNIR and SWIR respectively).

### 3.4. In-Flight Calibration Facility

If remote sensing instruments are to provide accurate data over the whole mission lifespan, their characterization and calibration must be an ongoing process that extends beyond the laboratory checks. An important part of instrument characterization is therefore resulting from the in-flight monitoring of instrument behavior over time. Stresses due to the positioning of the instrument within its carrier and due to changes in external temperature and pressure during flight, coupled with ageing-driven degradation, inevitably affect sensor characteristics. For the APEX instrument a built-in In-Flight Calibration (IFC) facility allows taking measurements before and after each image acquisition flight strip making use of secondary calibration standards [13]. Comparing IFC measurements taken in-flight with IFC measurements taken in the laboratory will allow assessing the stability of the instrument. If changes are such that the best detector performance cannot be guaranteed, the operational phase has to be terminated and APEX has to return to the laboratory for a fresh characterization and calibration [12], or to the manufacturer for an eventual upgrade of the instrument.

During IFC measurements a mirror will be moved into the optical path to reflect the light of the internal stabilized QTH (Quartz Tungsten Halogen) lamp through filter wheel openings into the APEX spectrometer.

**Figure 6.**
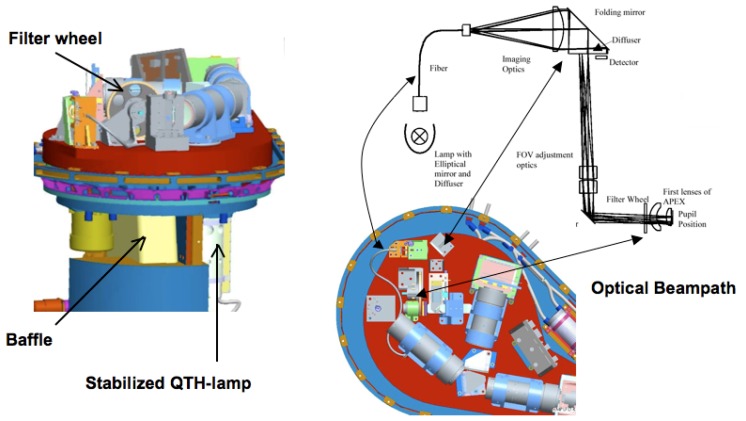
The In-Flight Calibration (IFC) facility.

**Figure 7.**
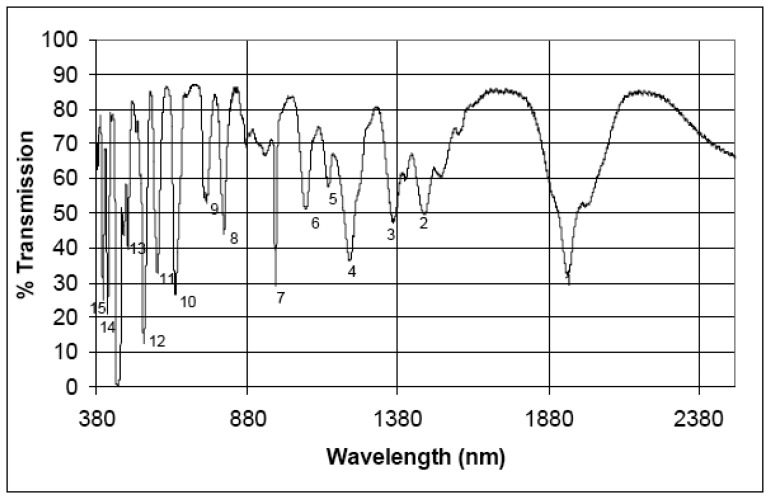
Spectral features of the NIST rare earth filter SRM (Standard Reference Materials) 2085 used in the IFC spectral calibration. Numbers 1 to 15 denote the spectral absorption features.

Five different filters are mounted on the rotating filter wheel (see Figure 6): a filter doped with rare earth material, three bandpass filters with small spectral bandwidths (at 694, 1,000 and 2,218 nm, respectively) and a NG4 attenuation filter used to avoid detector saturation at maximum radiance level in the VNIR channel. The sixth position on the filter-wheel is left empty with no filter inserted. The rare earth material filter from NIST (National Institute of Standards and Techonlogy) will be used to determine APEX spectral stability, i.e. to trace any shifts in center-wavelength position of the bands. This filter has been specifically manufactured for the calibration of hyperspectral instruments, exhibiting high spectral stability and a spectrum with many narrow absorption features through the visible and near infrared part of the electromagnetic spectrum (see Figure 7). The bandpass filters will be used in a similar fashion in order to monitor APEX spectral and radiometric stability [21].

The first APEX test flight, intended to verify the stability of spectral and radiometric parameters, has been performed in April 2008. The acquired data will be analyzed to gain knowledge of a number of other factors, such as geometric stability, co-registration between the VNIR and SWIR detector, dark current, influence of changing external pressure and temperature conditions with different flight heights.

The calibration data acquired through the IFC will be used to (a) provide quality metadata and (b) optionally generate correction coefficients that can be used for data calibration in the processing and archiving facility (PAF). Assimilation techniques will be developed to integrate the different calibration correction coefficients generated in the laboratory and in-flight, leading to improved quality of hyperspectral data products.

### 3.2. The Processing and Archiving Facility

The APEX processing and archiving facility (PAF) is hosted by VITO in the APEX Operations Center (AOC) at Mol, Belgium [22]. The APEX PAF is defined as the combination of all hardware and software components and their interfaces required for handling and processing APEX imagery and its related data [15].

The typical data size of hyperspectral imagery necessitates a computing architecture capable of delivering the needed processing power. The APEX PAF relies on the Master/Worker and Task/Data decomposition patterns implemented as a workflow framework [14, 23].

Major design requirements are on-demand, user configurable product generation, and full reproducibility of user orders and re-processing capability of any data product level. This is all made possible by the product and processing database (PPDB), which forms the heart of the processing system. The PPDB keeps track of (a) all imagery data, (b) related metadata such as calibration or housekeeping data and (c) subsequent products in the archive and stores the processing settings for on-demand generation of higher-level products. The PPDB is the single source for the dynamic building of the product order web pages.

The workflow automates the archiving of the raw input and its processing up to level 1C, thus generating a spectrally, geometrically and radiometrically calibrated, uniform data cube [24]. This sensor model inversion is parameterized by calibration cubes generated by the CTM.

Level 1C and higher-level products are ordered by user input via dynamic web interfaces. These orders are entered into the PPDB and trigger the processing by the workflow. The final data products are downloadable via FTP accounts.

### 3.3. Vicarious calibration

Vicarious calibration is an independent pathway for monitoring instrument radiometric performance, including error assessment with reflectance standards, field instruments and atmospheric radiation measurements. The experiment generally follows a reflectance-based approach with ground measurements of the atmospheric optical depth and surface reflectance over a bright natural target [25]. The accuracy of vicarious calibration experiments over land is highly dependent on the choice of an appropriate calibration target. Ideally, such a calibration site should be flat, bright, spatially uniform, and spectrally stable over time, near Lambertian for small angles off nadir, and of sufficiently large spatial extent. Desert playas (e.g., Railroad Valley Playa, NV, U.S.A.) are preferred for vicarious calibration due to their optical properties, predictably sunny conditions and low atmospheric aerosol loading [26]. In-situ sunphotometer data are used to determine aerosol model and horizontal visibility, subsequently applied for radiative transfer (RT) calculation. RT codes, such as MODTRAN-4 (Moderate Resoloution Atmospheric Transmission) [27] are used and often constrained by field data to calculate at-sensor-radiances. Input parameters to these codes include ground measurements of the surface reflectance, sun-target-sensor geometries and atmospheric properties (aerosol model, horizontal visibility). Reflectance-based vicarious calibration methods generally have absolute uncertainties of 3-5% [28]. In the past, RSL has performed extensive vicarious calibration efforts for MERIS (Medium Resolution Imaging Spectrometer) on ENVISAT (Environmental Satellite), where absolute uncertainties in the method were found between 3.36-7.15%, depending on the accuracies of the available ground truth data [29]. In the case of APEX, planned vicarious calibration experiments will account for a range of pre-defined flight altitudes and target radiances (bright and dark targets) to assess the sensor's radiometric performance.

## 4. Scientific products and application fields

**Figure 8.**
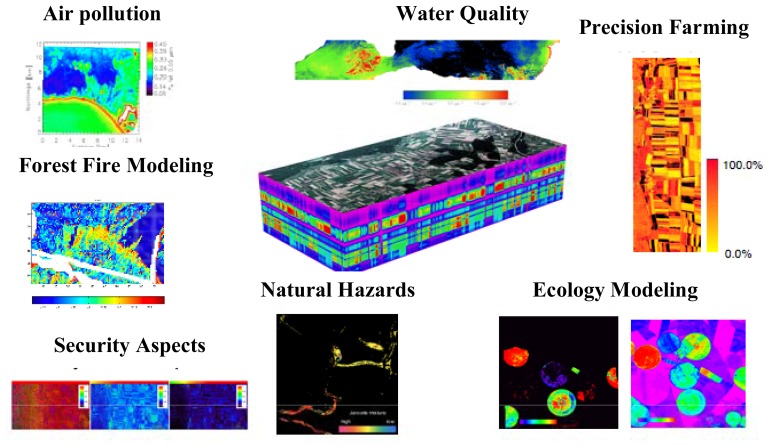
Example of APEX applications.

**Figure 9.**
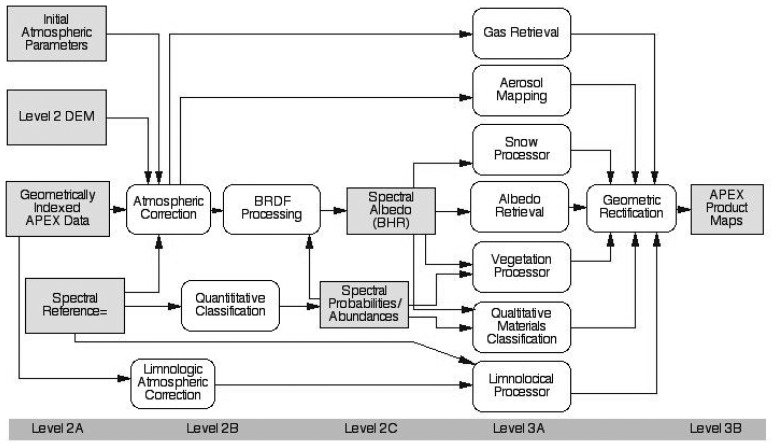
Level 2/3 APEX Processors.

Given the unprecedented performance requirements and data quality of APEX [22], the instrument will serve the needs of a broad range of both scientific and administrative user communities in Earth System remote sensing, e.g., in ecology, limnology, geology, atmospheric sciences, natural hazard and disaster management and materials detection. Applications based on APEX data will increasingly foster qualitative and especially quantitative remote sensing by allowing for improved Earth System variables retrieval (Figure 8) [30]. The optimized workflow for APEX Level 2/3 processing follows a product oriented way with major modules for the identified main hyperspectral applications [31]. These modules act as processors to deliver the expected products (e.g., plant biochemical distribution maps, inland water constituents maps, hazard maps etc.) following minimum standard requirements for optimized interoperability and processing within the APEX PAF. Application specific simulation models, empirical or physically based RT models, will form the basis of each module. Within the APEX Science Center (ASC) aiming at the scientific exploitation of APEX data, a number of application modules are presently being developed. Potential applications in the domains of water quality monitoring, vegetation analysis and ecology, aerosol retrieval, materials classification, snow characterization, as well as BRDF and spectral database issues are addressed. Future extensions to the modules and additional applications may easily be added to a streamlined level 2/3 workflow to support a growing number of researchers and data users (Figure 9).

### 4.1. Scientific data products

After the Level 0-1 processing of APEX data, well-calibrated at-sensor radiance data, scaled to a 16-bit format are available. At this stage, three options are distinguished, based on their respective levels of uniformity [24, 21]:

1. *Non-uniform data (Level 1A):* these data are containing the originally measured radiometrically calibrated data, without any corrections for smile and frown or co-registration. As such, no interpolation has been performed on the data except for bad pixel replacement. The data are of interest for highest resolution applications such as atmospheric sensing in the VNIR spectral range.
2. *Partially-uniform data (Level 1B)*: the specified quality of the APEX system defines small deviations regarding optical aberrations within each detector (i.e. below 0.2 pixels). When correcting for these smile and frown effects only, a set of detector-wise uniform data may be produced. Such data sets are well suited for applications making use of the spectral range of one detector only, e.g., geological applications in the SWIR or limnological applications in the VNIR.
3. *Fully-uniform data (Level 1C)*: co-registration (i.e. synchronization) [24] between the detectors is expected to be better than one pixel offset. Therefore, the creation of a fully uniform data set is feasible by interpolation of the SWIR detector outputs onto the spatial response of the (uniformized) VNIR detector. A spectral cut-off limit is defined between the detectors, in order to produce a contiguous spectrum across both detectors after interpolation. This level is expected to be the normal, and most requested output of the APEX calibration chain.

The Level 1 products are accompanied by geometric information, i.e., an index, which defines the orthometric locations of each pixel, which is produced on the basis of a DEM (Digital Elevation Model). In Level 1A, the index will refer to the reference centers of the pixels, in Level 1B, two indices will be required for the two detectors, respectively. The combination of Level 1 products with the geometric information leads to three kinds of *Level 2A* radiance products.

The subsequent compensation for atmospheric effects will use Level 1C or possibly 1B products. The atmospheric correction uses a special implementation of the ATCOR (Atmospheric Correction) procedure [32] to derive bottom-of atmosphere (BOA) reflectance, which may also be referred to as an in-field hemispherical-directional reflectance factor (in-field HDRF, [33]). This product is the (‘traditional’) *Level 2B* reflectance product, which is useful for methods relying on directional model inversion such as in vegetation canopy models.

The ultimate goal of radiometric compensation is to derive a directionally independent surface reflectance, i.e., a bi-hemispherical spectral albedo product, which we name *Level 2C* (Figure 9). Such a product allows an unbiased use of spectral processing techniques for classification and physical parameter inversion, as described below. A yet to be implemented BRDF correction method shall allow such processing in an automatic system.

### 4.2. Water quality monitoring

Natural inland waters contain a variety of optically active constituents, such as organic and inorganic suspended matter, phytoplankton and their pigments, and CDOM (Colored Dissolved Organic Matter). The relationship between the constituent's concentrations and the reflectance of water is non-linear, but can be described by their specific inherent optical properties (SIOP), i.e. scattering and absorption coefficients [34]. Reflectance is generally lower and less variable than with land surfaces, and atmospherically scattered radiance often dominates water reflected radiance in the blue region. Therefore, the quantitative determination of water constituents requires very accurate sensor calibration and atmospheric correction. In spite of this complexity, statistical approaches are applicable where extensive, concurrent ground truth measurements are available [35]. Physical algorithms based on radiative transfer modeling provide a more flexible alternative, such as the Neural Network-based MERIS case II water algorithm [36]. However, the time-consuming training of individual NN (Neural Network) for regional variations in SIOP is not very efficient for use with nonrecurring acquisitions with an airborne sensor.

The APEX level 3 water constituent product is based on the physically based modular inversion and processing scheme MIP [37]. MIP consists of an atmospheric correction module, which calculates subsurface irradiance reflectances from at-sensor radiances. Several retrieval modules exist for the calculation of aerosol optical thickness (AOT) based on multi-directional airborne measurements [37], on an atmospheric correction reference band in the water vapor window at 890 nm [38] or coupled water constituents and aerosol retrieval algorithms [39]. The inversion of subsurface irradiance reflectance into water constituent concentrations for regionalized SIOPs is then performed by a non-linear optimization procedure. A simplified process was automatized for MERIS data of Lake Constance, proofing the general applicability of this method and the adjustment of adequate SIOP [38].

### 4.3. Vegetation analysis and ecology

Vegetation is a key component of the terrestrial biosphere in terms of biomass production (food, fibre and fuel) and its role in land-atmosphere interactions. The properties of vegetation determine the exchange of energy and matter between terrestrial ecosystems and the atmosphere. Therefore, accurate characterization of vegetation properties and temporal dynamics serve as key components to many land-cover schemes that form part of Earth System models, ecosystem process models or water interception models, which in turn are used as prediction tools in climate and ecosystem research.

With the advent of imaging spectroscopy in the mid-1980s, a significant advancement was achieved in the modeling, monitoring and understanding of vegetation canopies due to the extended spectral dimension [40,41]. Recent imaging spectrometers have contributed significantly to the mapping of quantitative vegetation parameters [42, 43, 44], global change studies [45, 46], agroecosystem modeling [47] and precision farming [48, 49]. Lately, interest has arisen in using hyperspectral sensing for biodiversity monitoring [50, 51], ecological fingerprinting [52] and invasive species mapping [53].

The advanced data quality of the APEX instrument will progress many of these applications; moreover, it will inspire innovative combinations of advanced remote sensing products and foster developments of novel analyses techniques and applications. Together with further developments in radiative transfer (RT) modeling, APEX will help to derive a more robust and comprehensive characterization of the complex and dynamic nature of vegetation canopies, which serve as input to sophisticated Earth System and ecological models as well as decision making processes. However, only an integrated approach of remote sensing complemented with in situ sensing, through assimilation or modelling approaches, will allow a more consistent understanding of the relevant processes of vegetated ecosystems and the full Earth System. The linking between in-situ observations and coarse resolution satellite products can be substantially supported with APEX data by providing accurate, spatially and temporally comprehensive quantitative measurements of vegetation and land surface properties to overcome the spatial scaling gap at intermediate level.

### 4.4. Aerosols retrieval

Aerosols have a significant, yet largely unknown impact on the Earth's climate system. They are measured by sophisticated in-situ techniques and by remote sensing instruments from space. A gap remains between the local and the global scale. APEX has the ability to provide two-dimensional spatial data on aerosol properties, such as aerosol optical depth (AOD), Angstrom exponent, asymmetry factor and estimated particle size distribution. The aerosol retrieval benefits from its high spatial and spectral resolution as well as high signal-to-noise ratio (SNR).

The main objective of the APEX aerosol retrieval is to support the correction for atmospheric influences during the processing of APEX data to level 2B and above. Therefore, AOD and appropriate aerosol model information are needed. This step is crucial to generate high accuracy level 3 data. The secondary objective of the algorithm is to provide a high spatial resolution aerosol parameter map, which is of special interest to climate research, air quality monitoring and modeling purposes as well as for the validation of AOD products from satellite sensors.

**Figure 10.**
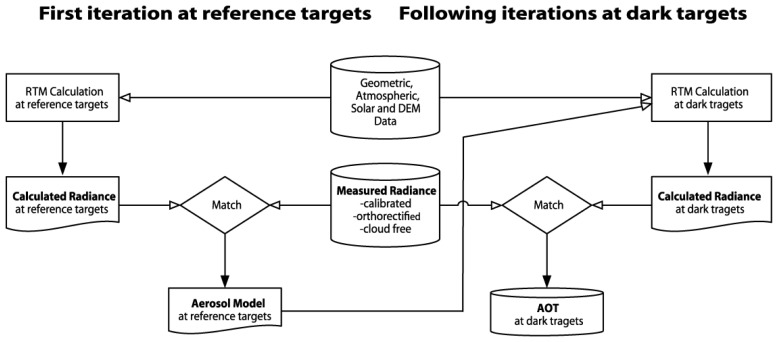
Aerosol retrieval algorithm flowchart. A first iteration is carried out at reference target pixels with a known spectral surface reflectance. This helps to constrain the unknown variables and to find the appropriate aerosol model. The following iterations continue with this aerosol model and retrieve AOD at dark pixels, where the influence of the error in surface reflectance is relatively small.

The aerosol retrieval strategy is explained in Figure 10 and [54]. The embedding of previous knowledge and reasonable assumptions on the atmospheric conditions are required to constrain the ill-posed problem of retrieval and solve it by means of inversion with a radiation transfer model. The available near-UV/blue spectral bands below 400nm are a further asset of APEX, which helps to reduce the influence of uncertainties by surface reflection assumptions. A recent study analyzed the sensor performance requirements for the AOD retrieval in terms of SNR and proved its feasibility with APEX for various surface reflectances [55].

### 4.5. Materials classification

Within the workflow for APEX Level 2/3, information extraction and classification is not confined to the major modules for dedicated variables retrieval, the so-called processors. Basic multiple threshold classification of at-sensor measurements into broad landcover classes (e.g. water, cloud, snow) is a prerequisite for the subsequent atmospheric correction and BRDF processing. However, the proper information and parameter extraction and the classification of landcover and materials are mainly based on the spectral at surface reflectances. In a first classification module, the hyperspectral reflectances are decomposed into a limited number of object primitives like quantitative fractions of (chemical) material components, considering the spectral database SPECCHIO (Spectral Input/Output) and further ground based measurements. The spectral quantification techniques applied are the Spectral Angle Mapper SAM [56], Linear Spectral Unmixing [57], Multiple Endmember Spectral Mixture analysis (MESMA) [58], Spectral Feature Fitting [59], Matched Filtering [60] and Mixture-Tuned Matched Filtering MTMF [61].

In a second step, quantitative fractions are incorporated in either the dedicated processors or are qualitatively classified in a labeling module. The high spatial resolution of APEX together with the fractional input restricts the labeling techniques for the latter module. Support Vector Machines SVM [62], artificial Neural Networks and SAM are supervised and pixel-based techniques, which can handle the fuzzy data space of fractional quantities and label the composition of materials in particular. The pixel wise techniques are applied to APEX land-data over Europe where variability within homogeneous landcover and land use classes is mapped, i.e. analysis of waste deposits, alpine geology, topsoil composition, vegetation states, etc.). Natural and anthropogenic landcover in rural and urban Europe are usually very heterogeneous and fractional. Pixel based labeling techniques will generally fail and are therefore replaced by object-oriented techniques, where object features include in addition to spectral and fractional quantities properties such as texture, shape, area, scale and neighborhood [63].

### 4.6. Snow characterization

Snow parameters such as snow grain optical equivalent diameter, impurities, liquid water content, snow-pack stratigraphy or variations in surface roughness are important input data for operational and scientific applications. Today most of these parameters are sporadically measured in situ at isolated locations and do not represent the small-scale snow-pack variations of Alpine regions [64]. Continuous large-area mapping of such parameters would both improve existing and foster new applications in the domains of hazard mitigation and climate change.

In the visible part of the electromagnetic spectrum snow has a high reflectance and is mainly sensitive to impurities. In the infrared part of the spectrum, snow absorbs most of the incoming radiance and is sensitive to a number of other parameters such as optical equivalent diameter (grain size), grain shape or liquid water content [65, 66]. Because of its high spatial, spectral and radiometric resolution, APEX is an ideal platform to a) deliver data for snow parameter retrieval, and b) identify the optimal sensor specifications for future remote sensing instruments designed to retrieve such parameters.

Rapid detection and mapping of recent avalanches is a promising application field. Information about avalanche occurrence is important for avalanche forecast, model evaluation and hazard map generation [64]. The turbulent transportation of snow in an avalanche mixes the layers of the snow pack and results in a reflectance different to the adjacent undisturbed snow. This feature could be mapped and measured by APEX. Information extraction from shadowed areas might still be feasible due to the instrument's high radiometric dynamic range, 14 bits for the VNIR channel and 12 for the SWIR. Furthermore, the good spatial resolution also enables the detection and mapping of small-scale avalanches.

### 4.7. BRDF

The BRDF (Bidirectional Reflectance Distribution Function) is an object inherent property and describes the dependency of an observed reflectance on the wavelength and the illumination and observation geometry [67]. BRDF effects can be readily identified in airborne and satellite imagery and do hinder the utilization of such data for subsequent analysis, as identical objects can appear to have differing spectral signatures. The severity of the BRDF effects in airborne imagery is dependent on the field of view (FOV) and on the orientation of the flight strip relative to the sun. Effects are most pronounced with large FOVs and flight directions perpendicular to the principal solar plane [68] and reach a maximum in the hotspot configuration (coinciding observation and illumination direction) for e.g. vegetation or in the specular reflectance configuration for e.g. water surfaces. The occurrence of both hotspot and specular reflectance is dependent on the solar zenith angle, terrain and the FOV of the sensor. Thus, for a given latitude and flat terrain only a limited number of across-track pixels are able to observe these effects. For example the minimum solar zenith angle in Zurich, Switzerland is roughly 23.9° on the 21 June, consequently, hotspot and specular reflectance would not appear in APEX imagery over flat areas. However, their occurrence can be expected for data acquired at latitudes ≤ 37° N.

BRDF effects are not necessarily undesirable artifacts that need to be corrected, but may also be considered to contain additional information for quantitative retrieval of e.g. vegetation [16, 69], snow [70] or soil [71] parameters. In both cases, fundamental knowledge of the BRDF involved may be needed for either correction or information extraction. The acquisition of the BRDF can be based upon hyperspectral data measured by a goniometer such as the dual-view FIGOS [17]. Such data sets can be used to analyse the anisotropic reflectance characteristics of objects and to retrieve the surface BRF (Bidirectional Reflectance Factor). Furthermore, expected BRDF effects for a specific sensor FOV, illumination direction and target type can be simulated based on goniometer data. The simulation consists of a spectral convolution and an observation angle selection according to the instrument's FOV specification.

**Figure 11.**
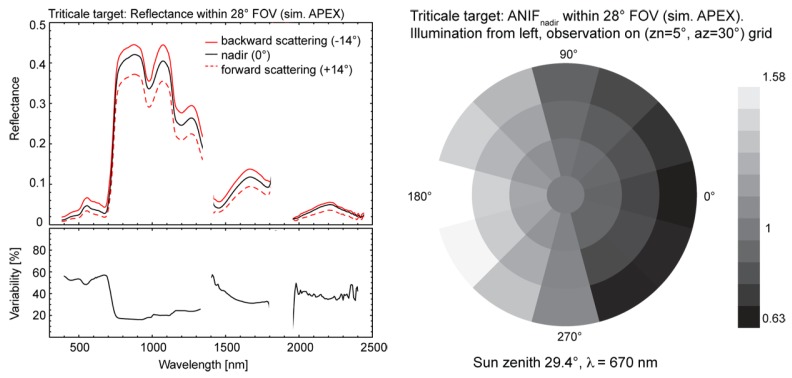
Simulated spectrodirectional signatures (left) and corresponding ANIF_nadir_ (right) for Triticale within the APEX FOV for an observation zenith (zn) angle of 5° and an observation azimuth (az) angle of 30°.

Figure 11 illustrates the spectrodirectional effects of a wheat target (Triticale) for a solar zenith angle (zn) of 29.4° simulated for APEX. The left plot shows the MODTRAN-4 simulated spectral signatures in the principal plane for nadir (0°), forward and backward scattering (±14°) and the directional variability (up to 60 %) as relative reflectance differences between the backward and forward scattering directions. The nadir normalized anisotropy factor ANIF_nadir_ for a FOV of 28° and a wavelength of 670 nm is presented in the right plot on an angular grid (Figure 11); the observation zenith (zn) and azimuth (az) angles are 5° and 30°, respectively.

For vegetation surfaces, strong BRDF effects are observed in the visible range of the spectrum, most prominently in the solar principal plane as shown in Figure 11. This is due to the distribution of shadowed and illuminated target facets, which leads to high reflectance values for backward scattering directions and a minimum for forward scattering reflectances. Directional effects are also visible in the NIR part of the spectrum, although at a lower degree (20 – 30% variability) due to increased multiple scattering processes.

### 4.8. Spectral Database: SPECCHIO

Hyperspectral applications as discussed in the previous sub-sections are often relying on spectral ground data and associated metadata. Such data are utilized to carry out feasibility experiments and parameterize processing modules for higher-level products in the APEX PAF. The organized storage of spectroradiometer signatures and describing metadata is a prerequisite for their efficient analysis and long-term utilization [72]. To these means the Remote Sensing Laboratories have developed the spectral database SPECCHIO [73]. SPECCHIO is used to (a) store spectral and metadata in a central repository which is accessible to all members of the laboratory, (b) serve as a spectral data source for various calibration/validation and simulation tasks and (c) provide parameters for APEX level 2/3 processing [31].

The system is comprised of a relational MySQL (Structured Query Language) database [74] and a graphical user interface implemented as a Java 2 application [75]. The Java technology keeps the software independent of the operating system, thus allowing its use in a heterogeneous computing environment.

Special focus has been put on the automated loading mechanisms to minimize the required user input. The generation of metadata in the system has been optimized by automated gleaning of metadata from spectral input files and containing data structures and by providing group updates on spectral sets [73].

Spectral datasets are retrieved by the means of metadata space queries, which put restrictions on metadata dimensions and thus create a subspace containing the required datasets [76].

RSL maintains an online version of the SPECCHIO database and interested parties can acquire a database account for testing and data sharing purposes. The SPECCHIO system installation package allows local installation and is intended for users requiring access control over their data. RSL distributes the SPECCHIO system package free of charge. For further information please refer to the SPECCHIO website: http://www.specchio.ch.

## 5. Conclusions

It took almost 15 years from the first ideas to the fully developed APEX system. Its design could be called conservative, but the specifications were such that, for instance new detectors had to be developed first, novel calibration concepts and a specific calibration laboratory had to be built up to ensure high data quality, and a fully fledged processing and archiving facility with its software and hardware had to be developed and installed.

The APEX Science Team has in parallel carried out research in some application fields with a variety of existing air and spaceborne sensors aiming at assessing the applicability of the new APEX system. Its great flexibility makes it an ideal universal platform for calibrating and validating existing sensors or simulating newly planned dedicated air and spaceborne systems. APEX is currently in its test phase. The near future will show for which of the anticipated roles the new instrument will be suited best.

## Figures and Tables

**Figure 3.**
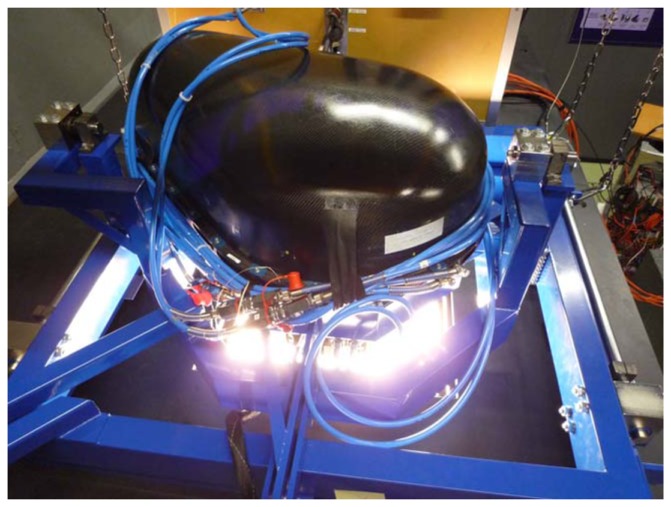
APEX installation on the integrating sphere at DLR for radiometric analysis.
